# Pheochromocytoma in Congenital Cyanotic Heart Disease

**DOI:** 10.1155/2018/2091257

**Published:** 2018-09-25

**Authors:** Carmen Aresta, Gianfranco Butera, Antonietta Tufano, Giorgia Grassi, Livio Luzi, Stefano Benedini

**Affiliations:** ^1^Department of Biomedical Sciences for Health, Università degli Studi di Milano, Milan, Italy; ^2^Endocrinology Unit, IRCCS Policlinico San Donato, San Donato M.se (MI), Italy; ^3^Department of Congenital Cardiology and Cardiac Surgery, IRCCS Policlinico San Donato, San Donato Milanese (MI), Italy

## Abstract

Studies on genome-wide transcription patterns have shown that many genetic alterations implicated in pheochromocytoma-paraganglioma (P-PGL) syndromes cluster in a common cellular pathway leading to aberrant activation of molecular response to hypoxia in normoxic conditions (the pseudohypoxia hypothesis). Several cases of P-PGL have been reported in patients with cyanotic congenital heart disease (CCHD). Patients affected with CCHD have an increased likelihood of P-PGL compared to those affected with noncyanotic congenital heart disease. One widely supported hypothesis is that chronic hypoxia represents the determining factor supporting this increased risk. We report the case of a 23-year-old woman affected with congenital tricuspid atresia surgically by the Fontan procedure. The patient was admitted to hospital with hypertensive crisis and dyspnea. Chest computed tomography revealed, incidentally, a 6-cm mass in the left adrenal lodge. Increased levels of noradrenaline (NA) and its metabolites were detected (plasma NA 5003.7 pg/ml, n.v.<480; urinary NA 1059.5 *µ*g/24 h, n.v.<85.5; urinary metanephrine 489 *µ*g/24 h, n.v.<320). The patient did not report any additional symptom related to catecholamine excess. The left adrenal tumor showed abnormal accumulation when 131I-metaiodobenzylguanidine scintigraphy was performed. A 18F-fluorodeoxyglucose positron emission tomography showed no significant metabolic activity in the left adrenal gland but intense uptake in the supra- and subdiaphragmatic brown adipose tissue, probably due to noradrenergic-stimulated glucose uptake. The patient underwent left open adrenalectomy after preconditioning with *α*- and *β*-blockers and histopathological examination confirmed the diagnosis of pheochromocytoma (Ki-67<5%). Screening for germline mutations did not show any genes mutation (investigated mutations: RET, TMEM127, MAX, SDHD, SDHC, SDHB, SDHAF2, SDHA, and VHL). Clinicians should consider P-PGL when an unexplained clinical deterioration occurs in CCHD patients, even in the absence of typical paroxysmal symptoms.

## 1. Introduction

Congenital heart disease (CHD) is a group of developmental abnormalities of the heart and great vessels whose incidence has considerably increased in the last decades. Cyanotic congenital heart disease (CCHD) represents a severe subset of CHD often characterized by neonatal systemic hypoxia. In CCHD a right to left shunt is observed and it results in deoxygenated blood entering the oxygenated limb of the vascular circuit. CCHD affects 1/1000 live newborns and represents approximately 10% of all CHD [1]. Early surgical treatment allows in most cases the reduction or elimination of chronic hypoxia.

Numerous case of congenital heart defects can cause Eisenmenger syndrome, including atrial septal defects [2], ventricular septal defects, patent ductus arteriosus, and more complex types of cyanotic heart disease. All these cardio-vascular alterations can lead to a more or less evident cyanosis with potential effects favoring the development of chromaffin cell alterations.

Pheochromocytoma and paraganglioma (P-PGL) are catecholamine-secreting tumors, which respectively arise from chromaffin cells of the adrenal medulla and the sympathetic ganglia. P-PGL are rare tumors representing about 5% of incidentally discovered adrenal masses [3]. Up to 35-40% of patients have disease-causing germline mutations [4] and the likelihood increases in young patients.

The coexistence of CHD and P-PGL has already been reported in previous studies and a causal link between the two conditions has been postulated [5, 6].

## 2. Case Presentation

We describe the case of a 23-year-old Caucasian female affected with congenital tricuspid atresia and intact ventricular septum. She had a history of palliative surgery since first days of life but her percutaneous oxygen saturation (SpO2) level remained around 80% even though a Fontan procedure was performed at 12 years of age. Persistent desaturation was related to the presence of venous collaterals between the Fontan circulation and left atrium.

**Figure 1:**
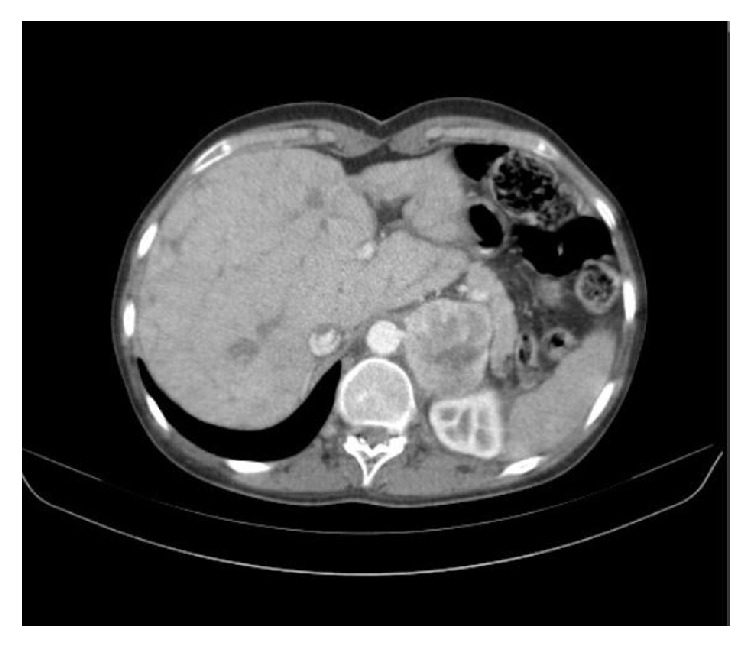
**Abdominal CT scan**: presence of a big mass in the left adrenal lodge (6-cm mass).

The patient admitted to Policlinico San Donato (San Donato Milanese, Italy) for hypertensive crisis, worsening dyspnea, and hemoptysis. There was no family history of relevant morbidities. On examination, her height was 175 cm, weight was 64 kg (BMI 17.7 Kg/m2), blood pressure (BP) was 160/85 mmHg, and SpO2 was 81% (room air). Electrocardiogram (ECG) showed sinus tachycardia (heart rate 101 beats/min), first-degree atrioventricular block (PR 220 msec), and right bundle branch block (QRS 140 msec). Chest computed tomography (CT) (Figure 1) incidentally detected a 6-cm mass in the left adrenal lodge.

**Figure 2:**
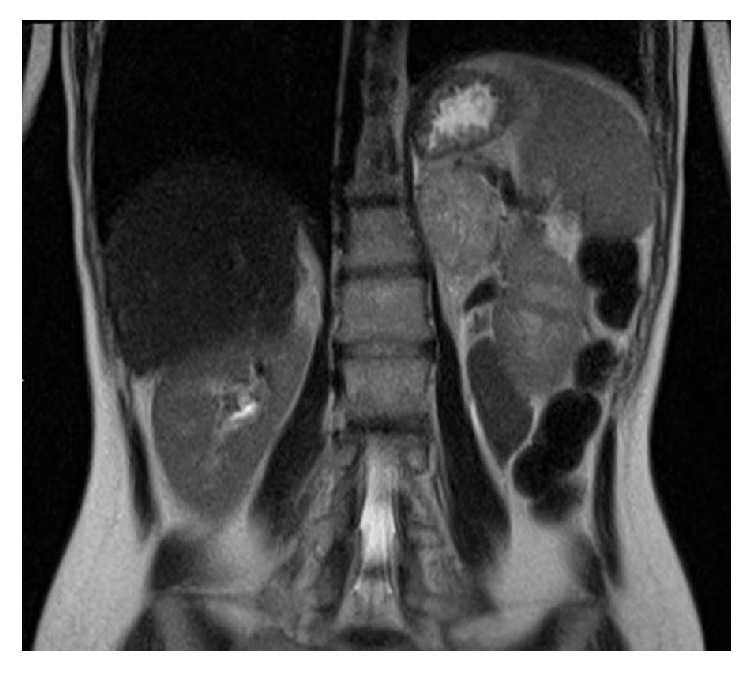
**Abdominal MRI scan**: presence of a big heterogeneous adrenal lesion, with hyperintense spots due to hematic content.

The presence of a heterogeneous adrenal lesion, with hyperintense spots due to hematic content, was confirmed by abdominal magnetic resonance imaging (MRI) (Figure 2).

Laboratory tests revealed increased levels of noradrenaline (NA) and its metabolites [plasma NA 5003.7 pg/ml, n.v. < 480 pg/ml; urinary NA 1059.5 *µ*g/24 h, n.v. < 85.5 *µ*g/24 h; urinary metanephrine 489 *µ*g/24 h, n.v. < 320 *µ*g/24 h; plasma adrenaline (A) 100 pg/ml, n.v.20-190 pg/ml; urinary A 15 *µ*g/24 h, n.v.1.7-22.4 *µ*g/24 h]. The patient reported no typical paroxysmal symptoms of catecholamine excess. Echocardiographic evaluation showed slight left atrial and ventricular enlargement, mild to moderate mitral regurgitation, and preserved systolic function (ejection fraction 65%).

**Figure 3:**
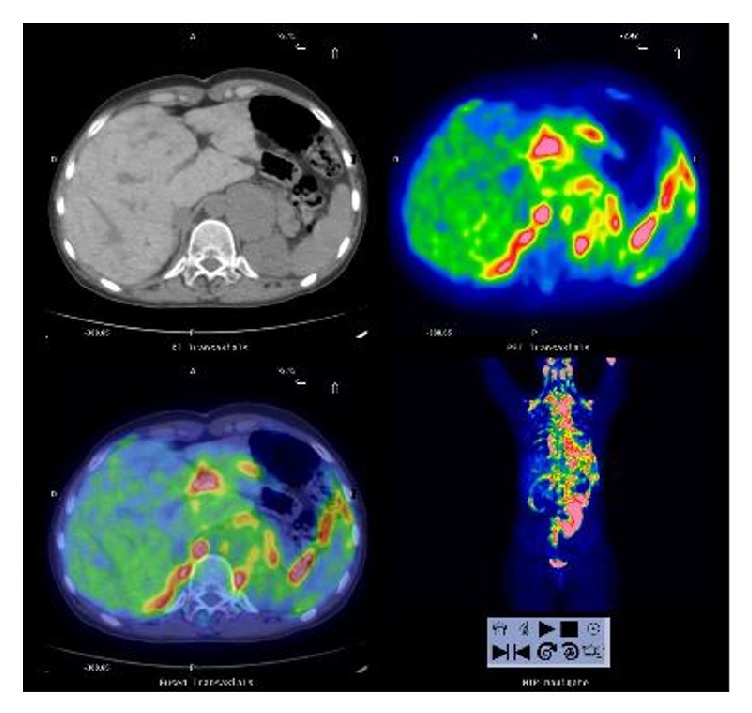
**Body 18F-fluorodeoxyglucose positron emission tomography scan**: no significant metabolic activity in the adrenal mass but intense uptake in supra- and subdiaphragmatic brown adipose tissue.

The diagnosis of pheochromocytoma was confirmed by 123I-metaiodobenzylguanidine (123I-MIBG) scintigraphy showing abnormal accumulation of radioactive tracer in the left adrenal gland. A 18F-fluorodeoxyglucose positron emission tomography (18F-FDG-PET) performed in order to exclude any extra-adrenal uptake: no significant metabolic activity in the adrenal mass but intense uptake in supra- and subdiaphragmatic brown adipose tissue was detected, likely due to noradrenergic-stimulated glucose uptake (Figure 3).

The patient underwent open left adrenalectomy after preconditioning with *α*-blockers (doxazosin) and, then, *β*-blockers (bisoprolol). Postoperative course was complicated by anemia due to hematoma formation in the left hypochondrium. Histopathological examination confirmed the diagnosis of pheochromocytoma with large hemorrhagic areas and scarce necrosis. No capsular or lymphovascular invasion was found. Immunohistochemistry revealed diffuse expression of chromogranin A, synaptophysin and neuron specific enolase, and S100 staining in sustentacular cells; Ki-67 was <5%. The P-PGL susceptibility genes VHL, RET, SDHA, SDHAF2, SDHB, SDHC, SDHD, MAX, and TMEM127 were analyzed for germline mutations and large deletions, via direct sequencing and multiplex ligation-dependent probe amplification methods; RET was only analyzed by direct sequencing. No aberration was found in these genes. Twelve months after surgery patient's BP and heart rate were under control and urinary NA and metanephrine levels were within the normal range. Plasma NA levels remained slightly increased (715 pg/ml n.v. 70-480), consistent with the hemodynamic changes in Fontan circulation [7].

## 3. Discussion

We present a case of pheochromocytoma in a young patient affected with congenital tricuspid atresia treated by Fontan surgery. Several cases of cooccurrence of pheochromocytoma and CCHD have been described in the literature [5]. It has been hypothesized that chronic hypoxia plays a fundamental role in these cases. In the last decades much evidence has been gathered supporting the role of hypoxia in P-PGL tumorigenesis. In 1973 Saldana et al. [8] documented a higher prevalence of carotid body paraganglioma in Peruvian adults living at high altitude in the Andes compared with those living at sea level, suggesting a link with chronic hypoxia. Glomus cells of the carotid body, such as chromaffin cells of fetal adrenal medulla, are specialized in sensing local oxygen tension in mammals [9] and can undergo anatomical changes if exposed to chronic hypoxia [10]. The hypoxia hypothesis has subsequently been supported since the 2000s by the discovery of the molecular basis of hereditary P-PGL. In fact a number of genes implicated in P-PGL syndromes, including succinate dehydrogenase (SDHx), von Hippel-Lindau (VHL), and hypoxia induced-factor 2A (HIF2A) genes, cluster in a common molecular pathway leading to the abnormal activation and stabilization of hypoxia-inducible factors (HIFs) in normoxic condition. This dysregulated accumulation of HIFs induces a number of downstream genes involved in angiogenesis, tumor growth, apoptosis, and energy metabolism. These findings have led to the hypothesis that chronic exposure to hypoxia in CCHD patients may increase the risk of developing P-PGL. In a recent letter to editor of New England Journal of Medicine Vaidya and colleagues report the identification of gain-of-function somatic mutations of EPAS1, which encodes for HIF-2*α*, in pheochromocytomas and paragangliomas in four of five patients who presented with cyanotic congenital heart disease. The authors concluded that the EPAS1 mutations endow chromaffin cells exposed to chronic hypoxia amplified the ability of development of the oncogenic properties of HIF-2*α* [11].

Opotowky et al. [12] showed that patients with CCHD have a greater risk of developing P-PGL [(odds ratio (OR) 6.0] whereas the OR in those with non-cyanotic CHD did not differ from that seen in patients without CHD. The same authors also pointed out that pheochromocytomas in CCHD patients share a number of clinical and biochemical features with pseudohypoxic PPGL syndromes, such as young age of onset, multiple tumors, and noradrenergic phenotype, suggesting a common pathogenetic molecular pathway. Unfortunately, in this case report, a complete assessment of all currently known genes involved in P-PGL syndrome has not been performed.

This case displays an exclusive noradrenergic phenotype and a young age of onset, suggestive of pseudohypoxic pheochromocytoma-paraganglioma syndromes [4]. The patient, although treated immediately after birth, had sustained prolonged cyanotic episodes in her life. Therefore, in the light of the above, the cooccurrence of CCHD (as well as in other numerous case of congenital heart defects that can cause cyanosis) and P-PGL in this patient could be explained by exposure to chronic hypoxia. This hypothesis was further supported by the absence of specific genetic background, often detectable in P-PGL young patients.

In conclusion, the combination of cyanotic congenital heart disease with P-PGL is uncommon but clinically relevant. The diagnosis of pheochromocytoma can be difficult in this clinical setting, as catecholamine excess symptoms (palpitations, arrhythmias, fatigue, dyspnea, and orthostatic hypotension) overlap with CCHD complications. Clinicians should consider P-PGL as a possible and potentially curable cause of otherwise unexplained clinical deterioration (in this case a slight hypertensive crisis and worsening dyspnea) in CCHD patients, even in the absence of typical paroxysmal symptoms.
